# CBM maze-scores as indicators of reading level and growth for seventh-grade students

**DOI:** 10.1007/s11145-017-9803-8

**Published:** 2017-11-18

**Authors:** Siuman Chung, Christine A. Espin, Claire E. Stevenson

**Affiliations:** 1 Department of Education and Child Studies, Leiden University, Wassenaarseweg 52, 2333 AK Leiden, The Netherlands; 2 Department of Psychological Methods & Statistics, Wassenaarseweg 52, 2333 AK Leiden, The Netherlands; 3 Department Psychological Methods, University of Amsterdam, P.O. Box 15906, 1001 NK Amsterdam, The Netherlands

**Keywords:** Reading growth, Secondary education, Progress monitoring, Curriculum-based measurement

## Abstract

The technical adequacy of CBM maze-scores as indicators of reading level and growth for seventh-grade secondary-school students was examined. Participants were 452 Dutch students who completed weekly maze measures over a period of 23 weeks. Criterion measures were school level, dyslexia status, scores and growth on a standardized reading test. Results supported the technical adequacy of maze scores as indicators of reading level and growth. Alternate-form reliability coefficients were significant and intermediate to high. Mean maze scores showed significant increase over time, students’ growth trajectories differed, and students’ initial performance levels (intercepts) and growth rates (slopes) were not correlated. Maze reading level and growth were related to reading level and/or growth on criterion measures. A nonlinear model provided a better fit for the data than a linear model. Implications for use of CBM maze-scores for data-based decision-making are discussed.

## Introduction

To be effective and productive in life, students must be able to read proficiently (Organisation for Economic Co-operation and Development [OECD], 2016), yet many students struggle with reading, even into adolescence. In 2015, 20% of 15-year old students in OECD countries (OECD, 2016) and 24% of eighth-grade students from the United States (U.S. Department of Education, 2016) could not read at basic levels of proficiency, meaning that these students had not mastered the reading skills expected of students their age. Such students face higher risks of poor educational and employment outcomes at age 19 and 21 (OECD, 2016).

Given the importance of reading, it would seem sensible to provide specialized reading instruction to struggling readers throughout their school careers, even into their secondary-school years. Specialized reading instruction for secondary-school students has been shown to be effective (Edmonds et al., 2009; Wexler, Vaughn, Edmonds, & Reutebuch, 2008), although effects sizes often have been small (Scammacca et al., 2016). Such small effects might be due in part to the magnitude and complexity of reading difficulties for secondary-school students (Vaughn et al., 2008). These students continue to struggle with phonological, fluency, and comprehension aspects of reading (Fuchs, Fuchs, Mathes, & Lipsey, 2000; Savage, 2006; Vellutino, Tunmer, Jaccard, & Chen, 2007), and the extent to which each aspect affects their overall reading performance differs among individuals (Catts, Adlof, & Weismer, 2006; Vaughn et al., 2008).

The magnitude and complexity of reading difficulties for secondary-school students creates unique challenges for educators as they strive to develop effective interventions for these students. Educators could be helped in their efforts if they were to have access to a tool that would be sensitive to small improvements over time, and could be used to evaluate the effects of specialized interventions for individual students. Such a tool would be of practical importance because, though effects may be small, minor improvements in reading during the adolescent years might translate into major improvements in success and satisfaction in adult life. A tool that is potentially suitable for monitoring the progress of secondary-school students with reading difficulties, and for evaluating the effects of interventions on an individual basis, is curriculum-based measurement (CBM; Deno, 1985, 2003).

### Curriculum-based measurement

CBM is a system designed for teachers to monitor the progress of students with learning difficulties, and to evaluate the effects of interventions on the students’ progress (Deno, 1985, 2003). CBM often is used within Multi-Tiered Systems of Supports (MTSS), and is uniquely suited for monitoring progress for ‘Tier 3’-students, that is, students who are in need of individualized, intensive instruction. CBM involves frequent (e.g., weekly) administration of short timed probes of equivalent difficulty. Scores from probes are plotted on progress graphs that depict student growth. Student growth is continuously compared to an expected (desired) rate of growth to determine whether the instruction should be changed or the goal raised. The ‘expected’ rate of growth is depicted by a straight goal line drawn on the graph that extends from the baseline to the anticipated level of performance at the end of the school year. Students are monitored frequently so that teachers can evaluate growth and make instructional decisions in a timely fashion. When teachers use CBM data to make instructional decisions, they affect significant improvements in student performance (Fuchs, Fuchs, & Hamlett, 2007; Stecker, Fuchs, & Fuchs, 2005).

The majority of research on CBM in reading has been conducted at the elementary-school level. In recent years more attention has been directed toward the secondary-school level (see Wayman, Wallace, Wiley, Tichá, & Espin, 2007, for a review), but that research has focused primarily on scores as indicators of performance rather than growth (Espin, Chung, Foegen, & Campbell, in press). To be used for instructional decision-making within Tier 3 settings, scores from CBM measures must be shown to be reliable, sensitive, and valid indicators of growth.

In this study, we examine the reliability, sensitivity, and validity of scores from CBM reading measures as indicators of growth for secondary-school students. We focus on one particular CBM measure: maze-selection. A maze is a passage in which every 7th word is deleted and replaced with a multiple-choice item consisting of the correct word and two distracters. Students read silently for 2–3 min, selecting the word that restores meaning to the text. The number of correct selections is counted and placed on the graph. To date, studies examining the technical adequacy of maze scores have produced tentative support for their use as indicators of reading progress for secondary-school students, but these studies have been limited in several respects.

### Research on CBM maze for monitoring progress of secondary-school students

Espin, Wallace, Lembke, Campbell, and Long (2010) and Tichá, Espin, and Wayman (2009) examined the technical adequacy of maze scores administered weekly over a period of 10 weeks. Participants were 31 (Espin et al., 2010) and 35 (Tichá et al., 2009) 8th-grade students who completed 4-min maze passages. Scores were the number of correct and correct-minus-incorrect choices for 2, 3, and 4 min. Criterion variables were scores on a state reading test (Espin et al., 2010; Tichá et al., 2009), reading group status, and scores and changes in scores on a standardized reading measure (Tichá et al., 2009).

Results were similar across the two studies, and provided support for the reliability, sensitivity, and validity of the maze scores. Alternate-form reliability coefficients ranged from *r* = .69 to .91, with the majority of coefficients above .80. Validity coefficients ranged from *r* = .75 to .86. Few differences in reliability or validity were found related to administration time or scoring procedures, with the exception that reliability increased somewhat with an increase in administration time. Scores were sensitive to (linear) growth, and growth on the maze was related to scores on the state reading test (Espin et al., 2010), to group status, and to growth on the standardized reading measure (Tichá et al., 2009).

Although the results of Tichá et al. (2009) and Espin et al. (2010) supported the use of maze scores for monitoring student progress in reading at the secondary-school level, there were limitations to the studies. The sample sizes were small and included only 8th-grade students. Monitoring occurred for only 10 weeks, and the same set of passages were used across the two studies. It was unclear whether findings from these two studies would generalize to a larger and more diverse sample, to monitoring across the entire school year, or to a different set of maze passages.

In 2012, Tolar and colleagues (Tolar, Barth, Fletcher, Francis, & Vaughn, 2014; Tolar et al., 2012) examined the technical adequacy of maze scores with a large sample (N = 1343) of students in grades 6 to 8 who were monitored across the entire school year using AIMS-web passages (https://aimsweb.pearson.com). Students in the study were monitored five times across the school year. Scores on the maze were the number of correct-minus-incorrect choices in 3 min. Reliability coefficients for maze scores ranged from *r* = .64 to .91. Validity coefficients ranged from *r* = .45 to .73 (Tolar et al., 2012). Scores reflected linear growth (Tolar et al., 2012, 2014), but not quadratic growth (Tolar et al., 2012). Finally, growth in maze scores was related to reading performance (Tolar et al., 2012, 2014), but not to growth on other reading measures (Tolar et al., 2014).

The results of the Tolar et al. (2012, 2014) studies provided further support for the technical adequacy of CBM maze scores for monitoring student progress, however, the study included only five measurement moments, too few for timely instructional decision-making within Tier 3 settings. It not clear whether the results from the Tolar et al. (2012, 2014) studies would generalize to the more frequent progress monitoring.

In sum, although the handful of studies that have been conducted to date at the secondary-school level have provided tentative support for the technical adequacy of maze scores for measuring progress in reading, there is a need for a large-scale study to examine the technical adequacy of scores from frequently administered CBM mazes. It would be important to examine whether earlier results replicate, and to more closely examine the relation between growth on the maze and growth on other reading measures. Whereas Tichá et al. (2009) found a significant relation between growth on the maze and growth on reading measures, Tolar et al. (2014) did not.

Finally, the present study should examine the extent to which CBM scores reflect linear growth over an academic school year. As described earlier, in practice, an assumption is made within CBM that students grow in a linear fashion across the school year. That is, the expected rate of growth is represented by a linear goal line extending across the school year. The line is used to make judgements about student progress and about the effectiveness of instruction on that progress. However, it is possible that growth is not linear. For example, research at the elementary-school level has shown that growth trajectories produced by CBM reading-aloud measures are nonlinear, with more growth seen in the first half than in the second half of the school year (e.g., Christ, Silberglitt, Yeo, & Cormier, 2010). In addition, studies demonstrate that the nature of growth in reading across school years is nonlinear, with more rapid growth in the first few grades, and less rapid growth in grades 3–8 (e.g., Kieffer, 2011). If the growth trajectories produced by weekly maze scores would prove to be nonlinear, it would have implications for the use of the data for instructional decision-making.

### Purpose

The purpose of the current study was to examine the reliability, sensitivity, and validity of scores from weekly administered CBM maze measures as indicators of growth in reading for secondary-school students. Multiple criterion variables assumed to represent students’ reading proficiency were included in the study, first because there is evidence that results vary widely across standardized reading tests (e.g., Cutting & Scarborough, 2006; Jenkins & Pany, 1978), and second, because the use of multiple measures allows us to examine whether evidence converges across various measures, building what Cronbach and Meehl (1955) refer to as a nomological network of evidence (Messick, 1989; see Espin & Deno, 2016, for a description specific to CBM). The study considers both linear and nonlinear (logistic) growth patterns to examine whether the assumption of linear growth underlying CBM implementation is warranted.

The following research question was addressed in the study: “What is the technical adequacy of CBM maze-scores as indicators of reading level and growth for secondary-school students?”. To examine this research question, three sub-questions were addressed:

1. What is the alternate-form reliability of scores on maze passages?
2. What is the sensitivity to growth of maze scores?
  - Do maze scores increase over time?
  - Do students show individual differences in growth trajectories?
  - Do students with higher initial maze scores show greater growth than students who start with lower maze scores?
  - What type of growth model, linear versus nonlinear (logistic), best fits weekly maze-scores?
3. Are maze scores valid indicators of reading level and growth?
  - Are maze scores and change in maze scores related to group status (school level and dyslexia status)?
  - Are maze scores and change in maze scores related to scores and change in scores on a standardized reading test?

## Method

### Participants

Participants were 452 7th-grade students (233 male) from three secondary-schools in the Netherlands. Mean age for the participants was 12.63 (SD = 0.63; range 12–15) years. Fifty-four participants were students with dyslexia. Dyslexia is defined in the Netherlands as a disorder characterized by a persistent problem with the learning and/or the application of skills in reading and/or spelling at the word level (Stichting Dyslexie Nederland, 2008).

Participants in the study were from a range of school levels. In the Netherlands, secondary-schools are organized into different levels, referred to (in order from lowest- to highest-level) as: practical, pre-vocational (low, intermediate, high), senior general secondary, and pre-university education (Dutch Ministry of Education, Culture and Science, 2005). Instruction and curriculum differ between school levels (i.e., students at higher school levels are required to process more complex information and perform more in-depth thinking than students at lower school levels). In reading, students at all school levels are provided instruction in which increasingly more complex texts are offered, but instruction is provided at a different pace. In addition, all school levels lead to differentiated criteria for graduation at the end of secondary school. For instance, pre-vocational students are expected to read expository passages about common topics either related or unrelated to their daily life, and to read simple narrative literature for adolescents on a surface level by the end of their secondary school, whereas, pre-university students are expected to read and understand a variety of expository passages about different topics within their curriculum and/or about socially relevant topics, and to be able to read and interpret narrative literature for adults by the end of secondary school (Dutch Ministry of Education, Culture and Science, 2009). In the year that the study took place, placement into school level was based on students’ academic performance during elementary school and on scores on a national achievement test.

Participants in our study often were placed in classes that combined school levels, thus for purposes of the analyses, we grouped students into three levels: low (practical and low pre-vocational), intermediate (intermediate and high pre-vocational), and high (senior general secondary and pre-university). Low achieving students were overrepresented in our study due to practical reasons.^1^ The distribution per school level in our sample versus the country was approximately 46.7 versus 14% for low, 36.5 versus 43% for intermediate, and 16.8 versus 43% for high levels (CBS StatLine, 2015).

#### Reading instruction

All participating schools provided reading instruction to their students using a reading-comprehension curriculum called *Nieuwsbegrip* (Understanding the news, CED-groep, 2012). The reading instruction follows a recursive process of 6 weeks, in which each week one of five reading strategies (i.e., predict, clarify, summarize, generating questions, and making connections) is discussed and applied to an expository reading passage. At week six all strategies are applied to a reading passage (CED-groep, 2012). It was not observed how much time was spend and what the quality of the instruction was.

### Instruments and procedure

#### Maze

The predicted variable in the study was the number of correct selections on the maze. Mazes were reading passages in which the first sentence was left intact, and thereafter, every seventh word was replaced by a multiple-choice item. Each multiple-choice item contained the correct word and two distracters. Distracters were within one letter in length to the correct choice and were clearly incorrect, that is the word did not (a) fit contextually in the text, (b) rhyme with the correct choice, or (c) sound or look like the correct choice (Fuchs & Fuchs, 1992).

The mazes were administered weekly using an online program developed by the researchers called *Mazesonline* ^®^ (http://www.mazesonline.nl). To complete the maze, students read silently through the passage, and selected a word at each multiple-choice item. After 2 min the task automatically stopped and the number of correct and incorrect choices were registered. The number of correct maze choices (CMC) were used in the current study.

Mazes for this study were constructed from expository reading passages of approximately 400 words. Passages were long enough to ensure that students did not finish the passage before time was up. Reading passages were written by the research team, and focused on general topics thought to be appropriate for and of interest to secondary-school students. Passages were equivalent in terms of scores on a common reading index used in the Netherlands (van den Berg & te Lintelo, 1977). This index is based on the average number of words in each sentence and the average number of syllables per word. The index level for the passages fell within the range of 69–73, a level considered comparable to the reading level of an average performing 5th-grade student at the end of the school year.

Reading passages were converted into maze passages. Multiple-choice items were placed between brackets and in bold. Passages were formatted so that all choices were on one line. The correct option was randomly placed in the first, second or third position for each item. To examine the suitability of the passages two small pilots were conducted, one with nine secondary-school students not participating in the study, and one with eight graduate students. Based on the results, passages that were too difficult (i.e., relatively small number of words read) were removed from the passage set. A set of 15 maze passages remained to be used in the study. These 15 passages also showed a distribution in scores among 127 7th- to 11th-grade low performing students (i.e., practical education) in an unpublished study conducted prior to the current study, indicating that the passages were not too difficult for the lower performing students. For the current study, 17 passages were needed. Thus, two additional maze texts were written at the same reading index level as the other passages. Information gleaned from the development of the other passages was used to write the two additional passages.

#### Reading proficiency

In line with Messick’s (1989) approach to establishing construct validity for maze scores as indicators of reading proficiency, we examined the pattern of relations between scores from the maze with various measures assumed to represent reading level and reading growth. These measures included group status (school level and dyslexia status) and scores on the standardized reading test: CITO-VVO. School level and dyslexia status have been already described, thus we only describe the CITO-VVO in the following section.

#### CITO-VVO

The *Cito Volgsysteem Voortgezet Onderwijs* (Cito Progress Monitoring System for Secondary Schools [CITO-VVO]; Cito, 2010) is a nationally-normed reading test in which students read 6–8 narrative and expository reading passages and answer 40–50 multiple-choice questions. It is administered once a year via pencil and paper. Different forms of the test are made for school level and grade levels. Scaled scores allow for comparison across school and grade levels and measurement occasion. The test for 7th-grade students was administered at the beginning and end of the school year, and given across two sessions at each measurement occasion. Administration time per session is 45 min (Cito, 2010).

Internal-consistency (Cronbach’s alpha) was reported to be α > .70. Construct validity included differences in school levels and discriminant validity: students in higher school levels performed higher than students in lower school levels, and CITO-VVO scores correlated with theoretical related constructs (i.e., vocabulary) and not with unrelated constructs (i.e., mathematics) (Egberink, Janssen, & Vermeulen, 2015a, b). Scores on the measure were obtained from the schools. In Table 1 the number of participants who completed the CITO-VVO per school level and descriptive statistics are provided.

**Table 1:** Descriptive statistics of the CITO-VVO

	*N*	*M*	SD	Min	Max
*CITO*-*VVO*					
Pre-test (Sept)	393	205.57	18.04	168	267
Low	166	192.93	11.77	168	221
Intermediate	152	207.14	11.44	182	245
High	75	230.39	12.69	206	267
Post-test (Jun/Jul)	386	211.16	29.48	148	309
Low	164	192.30	21.71	148	254
Intermediate	147	211.27	18.15	158	266
High	75	252.21	18.39	215	309

### Data collection

Data were collected between January and June of one school year. At the beginning of the study, teachers were informed about the purpose, background and instruments of the study. Teachers organized and supervised the electronic administration of the maze passages in *Mazesonline* ^*®*^ (http://www.mazesonline.nl). Students completed a total of 17 parallel maze passages, and received one maze weekly (with the exception of vacation weeks) over a period of 23 weeks between January to June. The research team was present for the initial administration to ensure that the system worked. During the first session teachers gave students a short introduction on the background of the study and instructions on how to complete the maze. Students then signed into the system and completed a practice task, after which they completed the first maze. Each week thereafter, students completed one maze via the online system.

### Data analyses

The first research question addressed the *alternate*-*form reliability* of maze passages. This was assessed via Pearson correlations in IBM SPSS Statistics 24.

To address the research questions related to the growth and validity of maze scores, multilevel analyses with maximum likelihood estimation were performed in the statistical software R, using the lme4 package (Bates & Maechler, 2010). Multilevel analysis is especially suitable for examining longitudinal data because it controls for dependence between measurements, missing data, and unequal groups in categorical variables (Hox, 2010). A multilevel model for linear growth consists of a within-individual level:

1 $$ Y_{ti} = \pi_{0i} + \pi_{1i}  \,maze\,session_{ti} + e_{ti} $$

where $Y_{ti}$ is the maze score for individual $i$ at time $t$. $\pi_{0i}$ is the maze performance for individual $i$ at the beginning of the study (intercept). $\pi_{1i}$ is the maze growth rate per week (slope) for individual $i$. $e_{ti}$ is the error term at the within-individual level.

The multilevel model also contains a between-individual level:

2 $$ \pi_{0i} = \beta_{00} + \beta_{01} Z_{i} + u_{0i} $$

3 $$ \pi_{1i} = \beta_{10} + \beta_{11} Z_{i} + u_{1i} $$

where $\beta_{00}$ and $\beta_{01}$ are respectively the mean intercept and slope to predict $\pi_{0i}$ from a between-individual level variable $Z_{i}$ (e.g., reading test score) for individual $i$. The $u_{0i}$ is the error term for the overall intercept $\pi_{0i}$, i.e., the difference between mean intercept and an individual’s intercept. The $\beta_{10}$ and $\beta_{11}$ are, respectively, the mean intercept and slope to predict $\pi_{1i}$ from a between-individual level variable $Z_{i}$ for individual $i$. The $u_{1i}$ is the error term for the overall slope $\pi_{1i}$, i.e., the difference between mean slope and an individual’s slope.

The multilevel model presented in Eq. (1) can be adapted to fit nonlinear growth as follows:

4 $$ Y_{ti} = \pi_{0i} + \pi_{1i}  (\ln (maze\,session_{ti} + 1)) + e_{ti} $$

In essence, Eq. (4) represents the same model as the linear model in Eq. (1), but the change of maze scores is expected to follow a logarithmic curve rather than a straight line.

To examine the *growth*, four steps were taken. First, the data were examined to determine whether the maze scores were sensitive for measuring growth in general. For this first step, a model with zero growth was compared to a model with a fixed effect for growth. Second, the data were examined to determine whether the maze scores were sensitive for measuring individual differences in growth between students (i.e., different growth trajectories), and compared to the model from step one. Third, a model in which the intercept and slope were correlated was included to examine whether students with higher levels of reading performance showed higher rates of reading growth compared to students with lower reading levels. This model was then compared to the second step model. The models were compared using a Likelihood Ratio Test (LRT), which was a Chi squared test that assessed change in goodness of model fit from a nested model to a more complex model containing additional parameters; a significant result indicated that the new model better fitted the data and that one could statistically infer that the additional parameters were of value (Hox, 2010). The analyses were performed for two growth models; linear versus nonlinear. Fourth, the two growth models were compared to determine which model best fitted the maze data. Because the LRT only can be used in nested models, the linear versus nonlinear models were compared by looking at the fit indices: AIC, BIC, Log likelihood and Deviance, where smaller indices indicated better fitting models (Hox, 2010).

Two approaches were used to examine *validity*. First, the relations between maze scores and group status (school level and dyslexia status) were examined. With regard to school level, we examined whether maze scores were higher and growth was greater for students at higher school levels than for students in lower levels. We applied a contrast forward difference coding to compare the three categories separately. With regard to dyslexia, we examined whether maze scores were higher and growth was greater for students without dyslexia than for students with dyslexia. The fixed effects of the linear and nonlinear models were interpreted at group performance levels. We examined whether the maze session, group level, and the interaction maze session X group level were significant predictors of maze scores.

Second, we examined the relations between maze scores (both performance levels and growth) and performance level and/or growth on the CITO-VVO. We examined whether the students who obtained higher scores and achieved greater growth on the maze also obtained higher scores and achieved greater growth on the CITO-VVO. The significance of the fixed effects was interpreted for the predictors maze session, reading test score or growth, and interaction maze session X reading test score or growth.

## Results

### Data inspection

Data were inspected,^2^ and scores were removed if students had a zero score due to the fact they made no selections, or had a potentially inflated score due to the fact they made random selections.^3^ We verified that the assumptions for multilevel analyses, normality and homoscedasticity at within- and between-individual levels were met. The intra-class correlation (ICC) was .70, which indicated that the variance explained at the between-individual level was high.

### Alternate-form reliability of scores on maze passages

Table 2 provides an overview of the correlations between scores on maze passages for adjacent administration weeks. The correlations ranged between *r* = .67 and .83.

**Table 2:** Correlations between Maze passages with 1-week intervals

	Passage comparison							
	0 and 1	1 and 2	2 and 3	3 and 4	4 and 5	5 and 6	6 and 7	7 and 8
*n*	150	95	169	154	214	65	106	174
*r*	.75**	.78**	.78**	.79*	.81**	.83**	.68**	.67**

	Passage comparison							
	18 and 19	19 and 10	10 and 11	11 and 12	12 and 13	13 and 14	14 and 15	15 and 16
*n*	198	59	245	172	134	139	163	95
*r*	.73**	.72**	.77**	.69**	.76**	.75**	.74**	.69**

**p < .01

### Growth of maze scores

The growth of maze scores was examined by fitting different models for linear and nonlinear growth.^4^ Table 3 presents the fit indices per model, and the Likelihood-Ratio-test results for each model comparison, and Table 4 (last row) presents the estimated mean initial maze scores (intercept) and progress (growth) for both growth models.

**Table 3:** Fit indices and LRT results for null, linear and nonlinear growth models

Model	Nested model	Fit indices				Likelihood ratio test (LRT)		
		AIC	BIC	–LL	Deviance	χ^2^	*df*	*p*
Null model								
M0		28,130	28,149	14,062	28,124			
Linear								
M1A	M0	27,961	27,986	13,976	27,953	171.21	1	< .001
M1B	M1A	27,859	27,891	13,924	27,849	104.4	1	< .001
M1C	M1B	27,858	27,897	13,923	27,846	2.37	1	.12
Nonlinear								
M2A	M0	27,908	27,934	13,950	27,900	223.65	1	< .001
M2B	M2A	27,800	27,832	13,895	27,790	110.14	1	< .001
M2C	M2B	27,800	27,838	13,894	27,788	2.50	1	.11

*M0* zero growth, *M1A/M2A* mean growth,* M1B/M2B* individual differences,* M1C/M2C* correlation intercept and slope, *AIC* Akaike information criteria, *BIC* Bayesian information criteria, *LL* log likelihood

**Table 4:** Mean maze intercept (initial performance level) and mean maze slope (rate of growth) per school level and growth model

Maze scores	*N*	Linear model				Nonlinear model			
		Intercept (π_0_)^a^		Slope (π_1_)^a^		Intercept (π_0_)^b^		Slope (π_1_)^b^	
		*M*	SE	*M*	SE	*M*	SE	*M*	SE
School level									
Low	211	22.32	0.42	0.10	0.02	21.41	0.43	0.80	0.15
Intermediate	165	26.54	1.05	0.17	0.06	25.53	1.05	1.28	0.39
High	76	33.43	1.23	0.24	0.07	31.19	1.22	1.99	0.43
Dyslexia									
Yes	54	21.30	1.37	0.15	0.06	20.33	1.35	1.16	0.42
No	398	26.39	0.35	0.15	0.02	25.34	0.35	1.19	0.11
Total	452	25.72	0.36	0.16	0.01	24.70	0.38	1.22	0.08

^a^See Eq. 1

^b^See Eq. 4

**Fig. 1:**
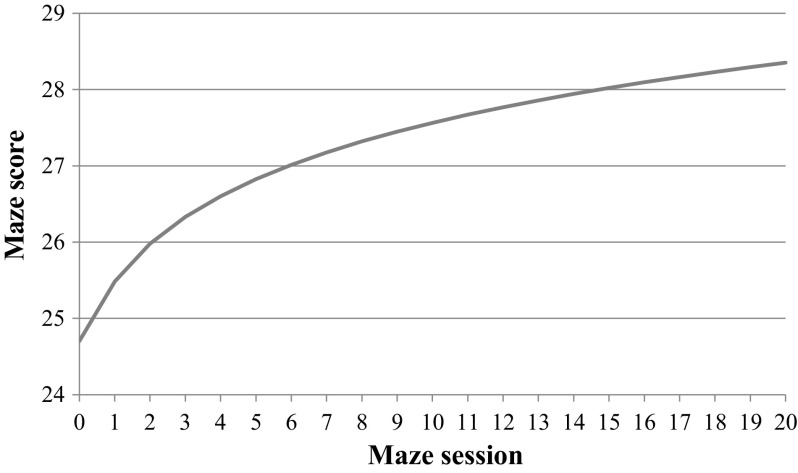
The estimated mean growth curve in the nonlinear model

To measure whether students’ maze scores *improved over time*, a linear and nonlinear (logistic) growth model were fitted to the data and compared to a null model where no growth was assumed (M0). For linear growth, the parameters of Eq. (1) at the within-individual level and (2) at the between-individual level were estimated; $\pi_{1i}$ was a constant. For nonlinear growth, Eq. (4) was used at the within-individual level. A linear mean growth model (M1A) was found to be a significantly better model than the null model, indicating that, on average, students changed over time on maze scores. The mean intercept was 25.72 correct choices, and mean growth was an increase of 0.16 correct choices per week. Students made on average 25.72 CMC during the first session, and after for instance ten weeks increased with 1.60 CMC to 27.32 CMC. A nonlinear growth model (M2A) was also found to be a significantly better model than the null model. The parameters of Eq. (4) were estimated and resulted in the formula: 24.70 + 1.22 × ln(maze session + 1); see Fig. 1 for a depiction of the nonlinear mean growth rate on maze scores. Students made on average 24.70 CMC during the first session and the increase of scores became smaller with each week. For instance, after 5 weeks, students made on average 26.89 CMC (an average increase of 0.44 per week), whereas after 10 weeks, students made on average 27.63 CMC (an average increase of 0.29 per week).

To measure whether students showed *individual differences in growth trajectories* on maze scores, random growth rates were added to the fixed growth model resulting in M1B and M2B for linear and nonlinear growth respectively (see Table 3). In both cases, the parameters of Eq. (3) at the between-individual level were also estimated. For both growth models, students showed significantly different growth trajectories. Thus, students’ individual growth rates on the maze differed.

Next a model in which the initial maze scores (intercept) and maze growth (slope) were correlated was fitted to examine whether students with higher initial maze scores grew more over time than those with lower initial maze scores (see Table 3). Initial maze scores were not significantly related to the rate of growth, indicating that students with higher initial maze scores did not show greater growth than students with lower initial scores.

Comparing fit indices (AIC, BIC, –LL, and Deviance; smaller is better, Hox, 2010) revealed that the nonlinear growth model was a better fit than the linear growth model for the students’ maze scores. Thus, the students’ growth on the mazes was better represented by a nonlinear than a linear model.

### Validity of maze scores

To address the research questions related to validity, relations between maze scores (both level and growth) and group status, and between maze scores and scores on the CITO-VVO were examined for both linear and nonlinear growth models. The difference (growth) score on the CITO-VVO was calculated by subtracting the pretest score from the posttest score. In Table 5, an overview of the estimated parameters of the fixed effects and its significance for both growth models is presented.

**Table 5:** Fixed effects and significance of group status and reading tests for linear and nonlinear growth models

	*N*	Linear			Nonlinear		
		*B*	*SE*	*t*	*B*	*SE*	*t*
School level							
Maze level: low versus intermediate contrast	452	− 6.36	0.61	− 10.42***	− 6.13	0.60	− 10.24***
Maze level: intermediate versus high contrast	452	− 9.19	0.79	− 11.62***	− 8.85	0.78	− 11.35***
Maze growth: low versus intermediate contrast	452	− 0.09	0.03	− 2.88**	− 0.70	0.20	− 3.49***
Maze growth: intermediate versus high contrast	452	− 0.10	0.04	− 2.62**	− 0.97	0.26	− 3.67***
Dyslexia							
Maze level: dyslexia	452	− 5.07	1.02	− 4.98***	− 5.01	1.00	− 5.02***
Maze growth: dyslexia	452	0.004	0.05	0.08	− 0.03	0.31	− 0.11
CITO-VVO							
Maze level: CITO-VVO level	393	0.24	0.02	15.00***	0.23	0.02	14.83***
Maze growth: CITO-VVO level	393	0.002	0.001	1.91	0.02	0.01	2.88**
Maze level: CITO-VVO growth	386	0.11	0.02	5.99***	0.10	0.02	5.71***
Maze growth: CITO-VVO growth	386	0.002	0.001	2.71**	0.02	0.01	3.40***

*** *p* < .001; ** *p* < .01; * *p* < .05

#### Group status

Mean group differences were examined for maze performance level and growth for more and less proficient groups, as defined by both school level and dyslexia status. School level was significantly related to both performance level and growth on maze for the linear and nonlinear growth model (see Table 5). Examining contrasts revealed that there were significant differences in performance level and growth for students at adjacent (low vs. intermediate vs. high) school levels. Students at higher school levels obtained higher maze scores and displayed steeper growth rates than students at lower school levels, see Table 4 for mean initial scores and growth rates per school level.

Dyslexia status was found to be significantly related to performance level, but not to growth on maze (see Table 5). This result was found for the linear and nonlinear growth model. Students with dyslexia performed lower on maze scores than students without dyslexia; however, the two groups did not differ in growth rates, see Table 4 for mean initial scores and growth rates per dyslexia status.^5^

#### CITO-VVO

The relations between maze performance level and growth, and performance level and growth on the CITO-VVO was examined. The scores were centered for easier interpretation of the fixed effects. The parameter estimates and the significance of the relations are reported in Table 5. Maze performance level was significantly related to performance level on the CITO-VVO for both growth models. Students with higher scores on the CITO-VVO had higher initial maze scores than students with lower scores on the CITO-VVO, see for example the results for an average score of 206 versus a high score of 260 on the CITO-VVO in Table 6. Students with higher scores on the CITO-VVO also showed higher growth rates on the maze than students with lower scores on the CITO-VVO. However, this relation was not significant in the linear model, whereas it was significantly related in the nonlinear model.

**Table 6:** Maze intercept (initial performance level) and maze slope (rate of growth) per growth model for CITO-VVO average and high scores and growth rates

	CITO-VVO level		CITO-VVO growth	
	Average (206)	High (260)	Average (5)	High (20)
Linear				
Maze level	26.42	39.25	25.75	27.35
Maze growth	0.15	0.24	0.13	0.17
Nonlinear				
Maze level	25.30	37.82	24.77	26.27
Maze growth	0.28	0.50	0.26	0.32

Both maze performance level and growth were significantly related to CITO-VVO growth, and this was the case for both linear and nonlinear growth models. Students who showed steeper growth on the CITO-VVO had higher initial maze scores and steeper maze growth than students with a lower growth rate on the CITO-VVO, see for example the results for an average growth rate of 5 versus a high growth rate of 20 on the CITO-VVO in Table 6.

## Discussion

Results of the current study supported the reliability, sensitivity to growth, and validity of maze scores as indicators of reading level and growth for secondary-school students.

### Reliability of maze passages

The alternate-form reliability of 2-min maze passages was moderate to moderate-good, with correlations ranging from *r* = .67 to .83. Reliability coefficients were slightly lower than those reported in Espin et al. (2010) and Tichá et al. (2009) where reliability coefficients were near *r* = .80, but were similar to those reported by Tolar et al. (2012). Both Espin et al. (2010) and Tichá et al. (2009) found that reliability coefficients increased with an increase in administration time. Thus for screening purposes, it might be wise to combine scores across two maze passages and/or to increase the length of the administration time to 4 min. For the purpose of measuring reading growth, the reliability coefficients can be considered acceptable to good; however, it will be important in future research to examine the effects of duration and schedules on the stability of the maze growth estimates (see e.g., Christ, Zopluoglu, Monaghen, and Van Norman, 2013, on CBM reading-aloud measures).

### Growth

The second research question addressed growth. We tested both linear and nonlinear growth models. In both cases, the models reflected that students improved over time, and that there were substantial individual differences in initial performance level and growth. These findings are consistent with findings from previous studies (Espin et al., 2010; Tichá et al., 2009). However, unlike previous studies, the linear growth rates in the current study were small: 0.16 correct choices per week compared to 2.17 for Espin et al. (2010) and 0.86 for Tichá et al. (2009).^6^ The obtained growth rates were similar to those found by Tolar et al. (2012) (see Footnote 6), where growth rates were 0.13 choices per week (although note that scores in that study were correct-minus-incorrect choices).

The inconsistent findings across studies might be due to several factors, including the composition of the sample, the nature and amount of reading instruction given to students, and the study design. With regard to the sample composition, both the current study and the Tolar et al. (2012) study had a relatively large proportion of low-performing students in their samples (47% for our study; 56% for Tolar et al., 2012). The percentage of low-performers in the Tichá et al. (2009) was 37%. In the Espin et al. (2010) study, performance levels were not reported, however, the mean score on the state reading test was similar to the mean score for all students in the state, indicating that there likely was not a disproportionate number of low-performing students in the sample. It will be important in future research to administer a validated set of maze passages to a large, representative sample of students at various grade levels in order to establish normative scores for both reading level and growth.

An additional reason for differences in growth rates might be the amount and nature of the reading instruction provided to the students participating in the studies. Previous research has demonstrated that under typical reading conditions, growth rates for struggling readers (i.e., students in special education) are lower than for non-struggling readers, but under optimal reading instruction conditions, growth rates for the two groups are the same (Deno, Fuchs, Marston, & Shin, 2001). Unfortunately, little information was provided in the studies regarding the amount and nature of the reading instruction provided to the participants. More research is needed into the effects of intensive, individualized reading instruction on the growth trajectories produced by maze scores.

A final reason for differences in growth rates might be the study design, more specifically, the duration, and schedule employed in the studies. Christ et al. (2013) found that the stability of growth trajectories produced by reading-aloud scores differed with duration (number of weeks) and schedule (weekly vs. biweekly data collection). Espin et al. (2010) and Tichá et al. (2009) collected data over a short duration (10 weeks) using a dense schedule (weekly), whereas Tolar et al. (2012) collected data over a long duration (school year) using a less dense schedule (every 6–8 weeks). In our study, we collected data over a relatively long duration (half a school year) using a dense schedule (weekly). The various combinations of duration and schedule might influence the precision of growth estimates. Research is needed to examine the effects of duration and schedule on the growth trajectories produced by the maze scores.

### Linear versus nonlinear growth

We found that maze reading growth was best represented within a nonlinear growth model. This result could be explained by the nature of reading growth. For example, Kieffer (2011) demonstrates a plateauing effect of students’ reading growth across school years, with rapid reading growth in the first few grades, and less rapid growth through grades 3 to 8. Although the results supported the use of a nonlinear growth model, the use of such a growth model produces a unique challenge to data interpretation. Within CBM, instructional decisions are made by comparing the student’s rate of growth to a goal line, which is typically represented with a linear growth line. Use of a logistic learning curve might better represent students’ reading growth, but it is likely to complicate data interpretation, and recent research has demonstrated that interpretation of CBM graphs can be difficult for teachers (van den Bosch, Espin, Chung, & Saab, 2017). A solution to this dilemma might be found in the use of electronic progress-monitoring programs. Given the current development and use of online and software programming possibilities, it is imaginable that programs could be developed that incorporate nonlinear long-range goals to enhance teachers’ ability to interpret and use progress data for instructional decision-making. However, it will be important in the future to examine the effects of using nonlinear growth models on the interpretation and use of the data.

A second factor to consider before recommending use of a nonlinear goal line relates to teachers’ expectations and the effects of these expectations on student growth. Although logistic growth curves might better represent students’ ‘typical’ reading trajectories, adoption of a logistic growth curve also might lead to less ambitious teacher expectations, and in turn, to less intensive instruction in the latter part of the school year. Research on CBM has demonstrated that when teachers have higher expectations and set more ambitious goals, students learn more (Allinder, 1995; Fuchs 1989). It may be especially important for students who struggle to maintain good quality reading instruction throughout the school year. If teachers are pressed to provide students the instruction they need, and to ignore possible slow incremental reading growth toward the end of the school year, it might lead to better student performance.

### Validity of maze scores

The third research question addressed the validity of maze scores as indicators of reading performance level and growth. We examined four types of relations: (1) maze performance level with performance level on criterion measures, (2) maze growth with performance level on criterion measures, (3) maze performance level with growth on criterion measures, and (4) maze growth with growth on criterion measures. On the whole, our results revealed that initial maze performance level and growth was related to performance level and growth on criterion reading measures. These results were consistent with what was found in previous studies (Espin et al., 2010; Tichá et al., 2009; Tolar et al., 2012, 2014). However, our result that growth on maze was related to growth on scores on the reading test was similar to the results of Tichá et al. (2009), but not to Tolar et al. (2014). These inconsistencies across studies might be explained by the fact that it is more difficult to statistically establish relations between more complicated relations (i.e., between two growth curves) as opposed to somewhat simpler relations (i.e., between growth and static scores or between static scores). The effects of more complicated relations could be influenced by several factors, including the chosen analysis method, the technical adequacy of scores on criterion measures and its sensitivity in detecting reading growth, or the assumption that reading growth is linear for the criterion measures (when using only pre- and posttest scores) (see, for example, McArdle, Grimm, Hamagami, Bowles, and Meredith, 2009, for a discussion on factors that should be considered when modeling growth data). There is a need to examine this issue more closely in future research.

### Limitations

One limitation of the study is that we administered the maze in the second half of the school year, so the results could only be based on the second part of the school year. Our purpose was to examine the technical adequacy of maze scores that could be used within a CBM framework in which frequent measurement is required. Such research is costly and time-intensive, thus the choice was made on first examining the technical adequacy of maze scores for a somewhat shorter period of time. A second limitation concerns the inclusion of a relatively large group of students with low proficiency. This means that our results best generalize to this population. This may also explain why the estimated weekly growth was rather low. Given that CBM is especially suitable for students who are in need of more intensive instruction, thus the low performing students, it is good that the results could be interpreted for this group of students. Future research should focus on a more representative sample administered throughout the whole school year to establish normative scores for maze reading level and growth. A third limitation is that no data was gathered on the amount and quality of the reading instruction. We recommend that further investigations into the technical adequacy of CBM maze growth rates also include data on the quality of reading instruction within the classroom.

## Conclusion and future directions

In conclusion, our results provide support for the reliability, sensitivity to growth and validity of maze scores for secondary-school students. Yet, several questions remain and need to be examined more closely in future research. A number of these points already have been raised throughout the discussion, including the use of a more representative sample, the examination of reading growth throughout the school year, and the inclusion of the quality of the provided reading instruction. In addition, future research should focus on how maze growth can be used for its intended purpose; that is, to examine if and how secondary-school educators use maze growth to evaluate the effectiveness of reading instruction and inform their instructional decisions for older struggling readers.

## Footnotes

1 One school with a large number of students in the high school-level agreed to participate, but had withdrawn their participation in the first few weeks of the study due to unavailable resources to collect the data.

2 For three maze passages, in the latter part of the passage the Mazesonline system registered the fact that the student had made *a* selection, but not *which* word was selected. Because the glitch occurred in the latter part of the passage, it affected only a small number of students. (Most students did not reach this point in the 2-min time limit.) For the students who were affected, we examined their scores on all other maze passages, and noted that they made few if any incorrect choices on their maze. We thus made the decisions to assume that their selections had been correct, and counted these items as correct choices.

3 There was the possibility that students could just randomly click on answers without reading the text, and thereby obtain an inflated correct maze score. To control for such random selections, we identified scores that included a larger than expected number of correct and incorrect choices. “Larger than expected” was defined as scores that were more than two standard deviations larger than the mean correct/incorrect group score for the passage. This comprised only 0.02% of the scores and these were removed from the analysis. Nine additional scores were removed because students did not make any selections. The removal of scores was considered not a problem, because only a short amount of scores were removed, and were from students from various proficiency levels.

4 We did not control for school in the analyses because our focus was on differences between school levels rather than differences between schools.

5 As suggested by one of the anonymous reviewers, we further unpacked the results relating to dyslexia status and school level. More specifically, we looked at whether there was a three-way interaction between school level, dyslexia status and maze level and growth. There was no reliable interaction between the three. The results are available upon request.

6 To compare across studies, growth rates for 3-min maze probes were prorated into 2-min growth rates by multiplying the 3-min growth rate statistic with 0.67.
